# Book Reviews

**Published:** 1827-05

**Authors:** 


### CRITICAL ANALYSES.

Quæ laudanda forent, et quæ culpanda, vicissim

Illa, prius, cretâ; mox hæc, carbone, *notamus.*—PERSIUS.

*On the Treatment of the more Protracted Cases of Indigestion.* By A. P. W. PHILIP, M.D. F.R.S. L. & E. *Being an Appendix to his Treatise on Indigestion.*—8vo. pp. 86. Thos. and Geo. Underwood, London, 1827.

THE title of this volume sufficiently shows its general object,—that of an Appendix to the work on Indigestion by the same author. The first paragraph which arrested our attention on perusing it was a notice of some animadversions on the opinions of Dr. W. PHILIP, by "two highly respectable physicians," who maintain that the distinctions drawn by our author, and consequently the principles of the treatment founded on them, are imaginary. The physicians alluded to are Dr. JAMES JOHNSON and Dr. PARIS; and, while we agree with them in the general estimate they have formed of the value of our author's doctrines, we are nevertheless willing to give him all the advantage which he can derive from pleading his own cause, which he does in the following words:—

"That at some period of indigestion the complaint, from having been a mere nervous affection, assumes an inflammatory character; that this tendency is the source of all the mischief which we sometimes observe in protracted cases; that the usual means do not relieve this state, but that it yields, in most cases, to very gentle means of a different description, are facts which, when once pointed out, I believe all who have frequent opportunities of treating the disease, who view its phenomena with accuracy, and have sufficient command of themselves to prevent the interference of preconceived opinions, cannot fail to observe; and in this opinion I am now supported by men of the greatest experience in our profession. It must therefore appear to me, that writers who do what in them lies to recall the former principles of treatment, as far as they have been superseded by what I believe to be more correct views, tend to do harm; and on this account, as well as in my own defence, I have thought it proper thus to state what I consider a sufficient reply to their observations.

"Dr. Paris, indeed, replies to himself; for he has been betrayed into contradiction in the most essential part of his subject. In the 240th and 241st pages of his Treatise, he does me the honour to say, 'I consider the train of reasoning by which Dr. Philip establishes the important fact, that long-continued irritation at length terminates in inflammation and organic derangement of the part affected, as constituting a very valuable part of his work.'

Yet, when I say that irritation of the digestive organs, surely inferior to no other, produces in its progress inflammatory tendency, and at length organic disease; and consequently that indigestion naturally divides itself into three stages, the stage of simple irritation, that of inflammatory tendency, and that of organic disease, he observes, in the 234th page, 'The arrangement is wholly artificial. Nature does not acknowledge it, nor will she submit to it!' The only thing difficult to account for is, that the disease should not sooner have been so divided." (P. 3—5.)

Now, with deference to Dr. W. Philip, we cannot perceive that there is any contradiction in this. It is a well-known pathological position, that long-continued disturbance of a part frequently gives rise to inflammation, and inflammation to disorganisation: but this is very different from the assertion, that indigestion "*naturally* divides itself into three stages," or that consumption of the lungs is caused "by long-continued disorder of the digestive organs."

When we first opened the volume, and found that our author thought it "equally due to the public and himself that some reply should be made," we must confess that we expected something more satisfactory than what we have met with. It is not enough to tell us, that he finds "nothing in their works but opinions brought in opposition to the facts" he adduces. This is merely begging the question. Suppose that phthisis pulmonalis does supervene upon indigestion. This is a simple *fact;* but it certainly is matter of *opinion* only that the previous indigestion has caused the subsequent pulmonary disease: it is a *post hoc ergo propter hoc* argument. We are satisfied that dyspeptic phthisis is a nonentity; and that the expression has been productive of serious evil, giving an idea of the disease which is theoretically incorrect and practically injurious.

The first practical discussion in the present Appendix relates to the Examination by Pressure on the regions of the Stomach and first Intestine. We are told that, while the pyloric region is always tender on pressure in the second stage of indigestion, that of the duodenum is only occasionally so. The part of the bowel, the examination of which is of most importance, is several inches below the pylorus, and about the same distance towards the right side from the centre of the body. The examination ought to be made in the erect position, as the viscera fall from the hand when the patient lies down. If pressure be made on both sides in corresponding situations, and with equal force, the left side will be represented as feeling "more free" than the right; and there is, besides, a sense of obstruction in the latter. A

difference is also perceptible to the hand, the right side being fuller and firmer: this difference, Dr. W. Philip states, is not dependent on the circumstance of the liver being situated on this side. Now, when any decided difference between the two sides can thus be perceived, it is, according to our author, "always the effect of disease;" and he adds, that of late years he has regarded the degree of distention as the best measure of the extent to which the digestive organs are deranged.

"When the region of the pylorus is tender, we know that the second stage has commenced; that a general inflammatory tendency, greater or less in proportion to that tenderness, prevails in the system, the removal of which is necessary to recovery. When this tenderness has extended to the region of the duodenum, we know that the affection of the pylorus has extended to it; but this is not merely proportioned to the degree in which the digestive organs are deranged, but to that and the degree of inflammatory tendency in the particular constitution. The difficulty with which the duodenum empties itself, on the other hand, very accurately tells us the degree of languor which prevails in the digestive organs, which, compared with the other circumstances of the case, more or less regulates all our means; and so constant a symptom of protracted indigestion is morbid distention of the duodenum, that, without saying a single word to the patient, the physician may generally know, by laying his hand on the region of this intestine, even on the outside of the clothes, whether the case be recent or not." (P. 12.)

When the duodenum is thus habitually loaded, no ordinary cathartic gives relief, and it is only to be permanently cured by such means as produce healthy bile. Sometimes, though but rarely, the tenderness (not pain) is greatest on the left side; and, except when this has been of a transitory character, passing off in a few days, our author has always found it prove obstinate. This form of the disease depends upon different causes in different instances, but always upon one more unfavourable than that which gives rise to tenderness in the polyrus or duodenum. It may be connected with enlargement of the spleen, or of the left lobe of the liver; but neither of these are looked upon as so common as "the state of the pylorus extending to other parts of the stomach." In some cases, the extension of the tenderness across the stomach depends upon disease of the pancreas.

The next subject brought under our notice is the state of the Organs of Waste in Indigestion. The derangement of these does not supervene till what our author calls the second stage of the disease, as up to this period the evil is confined to the stomach alone, or to this along with the organs immediately connected with it; while, in the second stage, the whole system participates in the morbid condition.

"It is not very uncommon, in the second stage of indigestion, for the organs of waste to be more debilitated than those of supply, and for the patient, from this cause, to get full and bloated. He acquires what, in common language, is called an unhealthy kind of fat. Part of what ought to be thrown off by the skin and other excretories is retained, and contributes not a little to the distressing feelings which the patient experiences. When this has happened to a considerable degree, the thinning is often rapid on the organs resuming their due functions; but, even when this is not the case, the patient almost always becomes thinner in the first part of his recovery. As it advances, however, and the organs of supply begin to resume their proper functions, he begins to regain flesh,. and by degrees generally returns to the standard natural to him in health, and thus generally becomes fuller than he has been during the greater part of his complaint. The loss of flesh without the loss of strength in the early part of the treatment, I have found almost a certain sign of ultimate recovery." (P. 29.)

With regard to the passage just quoted, we would observe, that, although we can easily understand that a patient who has effusion into the cellular membrane may get thinner during his recovery, and in this way *apparently* lose flesh, yet we have never seen a case in which the *actual* loss of flesh was to be regarded as of favourable omen.

Our author pays much attention to the Pulse, and gives us the following theory of the *tightness* which he looks upon as so characteristic of this pathological condition:—"The tight pulse, indeed, which is always present in a greater or less degree at this period, constitutes itself a certain degree of feverishness, and, when considerable, is accompanied with all its essential symptoms. The vessels, in consequence of the continued irritation of the most sensible nerves of our frame, are excited to embrace the blood more strongly than in health: hence the tight pulse. Now this state, although a morbid one, tends for the present to support the strength; and we know, when in the extreme, will even give a preternatural degree of strength." (P. 31.)

As we cannot regard the above as any thing more than a mere hypothetical explanation, so neither can we give our assent to the following doctrine:—"Could we suddenly relieve the dyspeptic from the causes of irritation to which he has been so long subject, by at once removing his disease, he would feel a depression of strength till the nerves had accommodated themselves to the change. The tightened state of the circulation would be relaxed, and the effect of this would be increased by the secreting surfaces, which were bound up, beginning to separate more freely their various fluids, and also by the alimentary canal being less distended with flatulence and a collection of undigested food, which, however injurious, for the time gives tension, and therefore tone." (P. 32.)

With all our respect for our author, we cannot help thinking that he is rather too fond of paradoxes. Thus, we have the loss of flesh—a sign of recovery; the removal of disease—giving rise to depression; and a collection of undigested food—giving tone.

Another example of the same general principle, that curing the disease is not always the best way to restore our patient to health, is to be found at page 51:—" It sometimes, though rarely, happens that those who have long been accustomed to a certain degree of tightness in the pulse, cannot, even for years, be brought to bear one as soft as the perfectly healthy pulse; all the means which produce such a pulse occasioning in them a considerable degree of depression, often such as unfits them for all the active duties of life: yet they are capable of most of those duties, and, on the whole, enjoy a tolerable share of health, if some degree of tightness be allowed to remain in the pulse." (P. 51.)

In order to illustrate his idea still further, our author compares the condition above described to nervous fever, in which he says " there is no local weakness supporting the disease." We do not think the analogy happy: if by local *weakness* he mean *disease,* it is obviously a gratuitous assumption without proof, or rather an assumption in the face of proof; for dissection shows local disease in nineteen cases out of twenty, or ninety-nine out of an hundred. If by local weakness our author means something else than disease, then we profess not to understand the expression. Again, we hold that the analogy is in other respects against him; for, in fever, when the "tightened state of the circulation" is "relaxed," and when " the secreting surfaces, which were bound up, begin to separate more freely their various fluids," so far from expecting that the patient " would feel a depression of strength," we know by daily experience that the very reverse is the case, and that he feels increasing strength every hour from the date of these symptoms.

We are free to confess that we look up to Dr. W. Philip's theoretical opinions as the least valuable part of his work, and we shall therefore dwell no longer upon them at present, particularly as we are desirous of quoting at length his practical observations on the application of different medicines to the treatment of indigestion under its various forms, or stages.

*Of the Nitrate of Potash in Indigestion.*—Some saline medicine I consider essential in the second stage of indigestion, for reasons which have already been pointed out; and I have found none so beneficial as the nitrate of potash. I feel no hesitation in saying, on the one hand, that it enables us at this period to lessen the quantity of mercury; and, on the other, that increasing the quantity of the latter will by no means produce the good effect of combining it with this nitrate, to say nothing of the greater tendency of mercury to impair the strength.

The nitrate of potash is chiefly indicated when there is a tendency to an increase of heat in the evening, or during the night, and particularly to a burning in the hands and feet; and in such cases its good effects are both greatest and most quickly apparent; but they are not confined to such cases. When there is no increase of heat, and even when the temperature is below the healthy standard, if this be not the case in a considerable degree, I still find this medicine to add to the good effects of the alterative course, provided there is an evident tightness of pulse, when examined in the way pointed out in my Treatise on Indigestion: but in such cases it is generally proper to combine it with some warm medicine. Small doses of tincture of orange-peel, or the compound tincture of cardamoms, are those I have generally employed.

When I first made trial of this combination, I doubted whether the good effects of the salt would not be wholly counteracted by the warm medicines; but I soon found that this is by no means the case, and that the advantage derived from the former, as an alterative, is very little interfered with by the latter. Here, it is true, we do not obtain the cooling effect of the salt; it is combined with the tincture to prevent this effect. It is its effects on the vessels of the digestive organs, and on the extreme vessels in general, that is wanted, and which appears to be little, if at all, impaired by this addition.

It is generally, not always, as might be supposed, in those cases where the surface is most inclined to be cold, that the patient is most subject to depression of strength and spirits. Here warm medicines are doubly indicated, and the occasional use of ammonia, even in considerable doses, I have found very beneficial, and very little liable to interfere with the alterative effect of the nitrate. The effect of the latter, however, sometimes materially interferes with its effect. I have seen some in whom the languor of circulation and coldness of habit were such, that the chilling effect of the nitrate could not be counteracted by any stimulus it would be proper to employ. In such cases the nitrate must be abandoned. They are, however, comparatively rare.

In a still smaller number, from idiosyncrasy of constitution, even very small doses of this salt cannot be borne, apparently from the irritation of the stomach and bowels which they occasion; and I have met with cases in which none of the salts, into the composition of which potash enters, could be borne.

Although the cases in which it is necessary to abandon the nitrate of potash are rare, it is not very uncommon to be obliged to give it in small doses, five or six grains. It should never be given in such doses as very sensibly to add to the sense of depression. But such in general is the effect of this medicine in the second stage of indigestion, that it is not at all uncommon for patients, guided merely by their own feelings, to continue the use of it after they have gradually laid aside all others, and to declare that they derive from it a kind of relief which they never experienced from any other means. This has not been the observation of one or two, but of a large proportion of those who have used it. Yet nobody would think of giving nitrate of potash in the commencement of indigestion; and it is not even mentioned in the catalogue of stomachic medicines. Can any opinions be brought in opposition to these, and many similar facts, laid before the reader in my Treatise on Indigestion, tending to establish the same position,—that the nature of this disease is changed in its progress; and requires in its different stages very different, and even opposite plans of cure? Those who maintain such opinions, have a very imperfect knowledge of indigestion. Their knowledge of it has never gone beyond the first stage; from which their views of its nature, as well as plans of treatment, are derived; neither of which, a more careful observation will teach them, are applicable to the stage before us.

I have found the good effects of nitrate of potash sensibly increased, by combining with it a small quantity of mucilage, and a very slight anodyne. From six to twelve minims of tincture of hyoscyamus, or a combination of two or three drops of laudanum with four or five of wine of ipecacuanha, I have found the best. These doses will only appear trifling to those who have not attentively watched the symptoms of indigestion, in the more advanced stages of which the nerves, from repeated irritation, often acquire a sensibility which appears almost incredible. The gums are among the least sensible parts of our frame, but those who have been troubled with carious teeth know how exquisitely sensible they may be rendered by irritation of long continuance.

I have had occasion to observe that indigestion attends, and, I might have said, lays the foundation of most of the diseases of infancy; and it is remarked, in the Treatise to which this is an Appendix, that the duration of the first stage of indigestion is very various, the symptoms of the second stage showing themselves at various periods in different cases. In children, those of the second stage supervene very early, and the disease in them often appears to commence in the liver rather than the stomach, the latter suffering only secondarily, which is the reverse of what usually happens in the adult, at least in this country. It is the early supervention of the second stage which renders saline medicines so essential in their diseases.

If the disease has made any considerable progress in them, no course of mercurials, or any other means, will succeed well without medicines of this description; and I have found the nitrate of potash invaluable in most of their diseases. Their nerves, as well as vessels, are more irritable than those of the adult. It is on this account that in them the more advanced stages of indigestion supervene more readily, and are attended with more fever, and more apt to produce serious derangement. Continued irritation of the digestive organs, which in the adult produces a tight pulse, and often a tendency to increased heat, in them produces actual fever, which is only a greater degree of the same symptoms.

Such is the nature of what has been called the remitting fever of children, which is so apt, when neglected, to end in effusion on the brain, the part in children most liable to suffer from the general irritation kept up by a deranged state of the digestive organs. For once that the hydrocephalus of children arises from other causes, it arises twenty times from affections of these organs.

*Of Tartarised Antimony in Indigestion.*—Tartarised antimony is proper in many of the same cases in which nitrate of potash is so beneficial; but it is a medicine of very different properties, and the principles which regulate its employment are very different. It appears, from experiments which the College of Physicians did me the honour to publish in the last volume of their Transactions, that, of all the means that were tried, tartarised antimony had the greatest effect in suddenly exciting the action of the skin. It has comparatively little effect in exciting sensible perspiration; but, as appears from these experiments, as well as many other circumstances, it is not by sensible perspiration, but by a free state of the insensible action of the skin, that its vigour is indicated.

When the reader considers what has been said respecting the state of the skin in indigestion, he will be prepared for the good effect of such a medicine in the more protracted cases of this disease. I have had many patients who told me that they could always secure a good day by exciting sensible perspiration in the morning. This, for a reason just mentioned, and others stated in my Treatise, should not be our aim in indigestion; but it is more favourable than the arid state of the dyspeptic's skin, and affords temporary relief.

When the surface is dry and the tendency to feverish attacks considerable, and we have reason to believe that the disease is, in a great measure, supported by the general state of the secreting surfaces, the tartarised antimony, as might *a priori* be expected, is often a valuable medicine; and I was agreeably disappointed to find that doses so minute as neither to excite nausea nor any increased sense of debility, are often sufficient to produce a sensible improvement. A slight degree of nausea, if it be only occasional,

I have found of little importance, and, contrary to what might be expected, it seldom even impairs the appetite. The antimonial has always been laid aside when it has appeared to increase the sense of sinking. The dose I have employed has generally been from the tenth to the eighth part of a grain, three or four times a-day. I have never seen the least bad effect from such doses, even when continued for months; and the patient, when it was laid aside, missing its good effects, has often requested to be allowed to resume it.

Analogous to what takes place in fever, when the tendency to increased heat is greater than usual, it has been found particularly serviceable, combined with the nitrate of potash. But of all the cases in which it was employed, it was found most beneficial in those where the dry skin and debilitated state of the other excretories had produced a determination of blood to the head, and it has been found necessary to continue it in such cases, after all other medicines had been laid aside.

Even in the early periods of the disease, great advantage is often derived from combining small doses of tartarised antimony with cathartics. It frequently has an operation on the bowels analogous to its effect on the skin, relaxing the surface, and thus rendering the action of cathartics more free. The same observation, indeed, applies more or less to all the secreting surfaces. In this respect its operation resembles that of mercury, but it produces its effects more quickly, and they are not, as in the case of the latter, apt to accumulate in the constitution, which makes it a less powerful medicine, but renders it safer, and thus, under certain circumstances, increases its utility. It may sometimes, with great advantage, be substituted for mercury, and very often combined with it, for the purpose of rendering less mercury necessary.

The beneficial effects of antimony in cutaneous affections, has long been acknowledged; when indigestion produces such affections, therefore, it is doubly indicated.

The operation of the colchicum is in many respects analogous to that of antimony; I have often used it, in very minute doses, for the purpose of relaxing the skin and softening the pulse in the advanced stages of indigestion. It was not among the medicines whose effects were compared with those of tartarised antimony, in the experiments just referred to; I cannot, therefore, say whether, on the whole, it may be equal to that medicine as a diaphoretic. In some respects it bears the same relation to antimony that this medicine does to mercury. Its effects are more sudden and more transitory; but it is capable of more violent effects than either of those medicines, and must always be used with caution, except in very minute doses.

The colchicum often has a peculiar effect in relieving the local inflammatory affections, so apt to supervene in protracted cases of indigestion, particularly those of the head and chest, and rheumatic pains of the muscles. I have often been disappointed in this effect of the colchicum before evacuations, and seen it act like a charm after them. When employed only with a view to relax the pulse and excretories, I have used it in extremely small doses; in rather larger doses, with a view to relieve cough and pain; but always lessened the dose, if it produced more than a very gentle action on the bowels, or a decided softness of the skin.

In the second stage of indigestion, whatever plan is adopted, much depends on the gentleness of the effect produced. I have long been convinced that this state is only to be removed by a slight effect regularly kept up for a considerable length of time. All powerful means, which are necessarily transitory, because they would soon destroy the patient if they were continued, fail to cure, and very often aggravate it.

In the first stage, when the first is unimpaired, and the habit of the disease feeble, powerful means will sometimes at once check its progress. In the second stage, where the opposites of these conditions obtain, this never happens. It is by the most gentle and frequently repeated impressions that the organs are solicited once more to resume their healthy action.

From what has just been said of the effects of the colchicum, compared with those of antimony, the reader will perceive that the former is, on the whole, less suited to the second stage of indigestion than the antimony; although its more speedy operation, its peculiar effect in relieving the inflammatory tendency, and particularly the power it sometimes evinces of allaying pain and cough, renders it preferable under certain circumstances.

The effect of tartarised antimony in severe nervous agitation, is very remarkable. Its power, even in allaying the symptoms of mania itself, is well known. I have found rather larger doses than those just mentioned, combined with moderate doses of hyoscyamus, by many degrees the most powerful means of allaying the more severe forms of nervous irritation which now and then appear in protracted cases of indigestion, not depending on any local irritation, the seat of which the patient can point out, but on the state induced by long continued irritation of the general source of nervous power.

*Of Ammonia in Indigestion.*—The effects of ammonia in certain states of indigestion are very valuable, and such as cannot be produced by any other means. We have no other means which so powerfully excite the nerves with so little disturbance to other parts. My attention was called to it above twenty years ago, by the essential benefit derived from very large doses of it in a case which had resisted all the usual means.

In some cases of indigestion, with the contracted pulse of the second stage, the vital fluids seem, as it were, to leave the surface, which is obstinately cold. The pulse in such cases is always very feeble, and the patient, for the most part, complains of great depression, hangs over the fire, and says that no exercise he can take has the effect of warming him. The nerves here are failing in one of their essential functions, that of supporting, by their action on the blood, the due degree of animal temperature; for in all such cases the temperature, measured by the thermometer, is actually, and sometimes considerably, below that of health. Here the ammonia is invaluable, being less apt, than any other stimulus of the same power with respect to the nerves, to excite the heart and blood-vessels; which, from the tendency of the disease, are inclined to a degree of excitement beyond that in due proportion to the state of the other powers.

The carbonate of ammonia may be taken in doses of from five to ten grains several times a-day with safety, and probably in larger doses; and it rarely fails, if given in the proper dose, with such exercise as the patient can bear, to diffuse warmth throughout the system. Nor is the benefit derived from it of a mere transitory nature. A state of chill tends not only to aggravate all the symptoms, but to confirm the disease. I have even known the digestion constantly deranged by the temperature of the room being so low as to cause a feeling of chilliness.

Ammonia is also a valuable medicine in most of the nervous affections which attend indigestion, even when the patient is not particularly chilly, provided the nerves are so far languid in the function of preserving the temperature as to allow of its being taken in considerable quantity without heating too much; an inconvenience which attends the free use of it in most cases of the second stage of the disease.

I have little doubt that, to the tendency of ammonia to excite the skin, we must in part ascribe its good effects in indigestion. It is probably chiefly to this effect that we ought to ascribe the advantage derived from some of its preparations, particularly the liquor ammoniæ acetatis, in the second stage of indigestion; in which I have repeatedly seen it eminently serviceable, when the ammonia itself heated too much. Nothing, in this stage of indigestion, more requires the attention of the physician than adapting the tendency of the treatment to heat or cool, to the circumstances of his patient. If we here attempt to lay down any general rule, we shall be led into error. The peculiar circumstances of the disease or constitution, for reasons which we cannot always detect, bear the one or other tendency better in one case than in another. The state of the pulse, and the effect of the means employed, are our best guides; and, on our forming a correct judgment in this respect, both the comfort of our patient and his final recovery greatly depend.

These observations are as applicable to diet as medicine. I have elsewhere pointed out that there are cases of the second stage of indigestion, in which an abstinence from animal food is proper: such cases, however, are rare. But, when the pulse is obstinately tight, abstaining from it two days in the week, I have sometimes found to produce an effect which it is impossible to procure by any other means. The patient has felt himself almost immediately more at ease. The bowels in particular have become less irritable, and more obedient to medicine; the skin softer, and the countenance much improved, and the ammonia, or other stimulants of the nervous system, better borne. The patient is often so pleased with the effect, that he thinks he has found a sovereign remedy for all his complaints. Let a dyspeptic, whose pulse has not acquired the same degree of tightness, and whose skin consequently is softer, follow the same plan, and it will do little more than add to the debility, and increase the flatulence both of the stomach and bowels. On the other hand, the stimulant, which the latter will tell you saves him from despair, given to the former, will hardly fail to increase all his sufferings; and yet, to a superficial observer, these two patients are much in the same state. They both complain of depression of mind and body, and those thousand nameless symptoms which attend irritation of the nerves of the stomach and bowels, rendered doubly sensible by their long-continued irritation.

---

*Practical Observations on the Application of Lunar Caustic to Strictures in the Urethra; illustrated by Cases proving the Permanency of the Cure by that Treatment. To which are added, some Remarks on its Use in Strictures of the Œsophagus.* By M. W. Andrews, Member of the Royal College of Surgeons, London. Second Edition.—8vo. pp. 249; with Plates. Callow and Wilson, London, 1827.

It is exactly twenty years since this volume first appeared; and, although it was noticed soon after in this Journal, (vol. xviii.) yet the long period which has since elapsed places it before us rather as a new work, than as a new edition of an old one.

The author commences with some general remarks on stricture, and then more particularly alludes to the opinions of Sir E. Home, as detailed in his third volume. He observes, that if the description given by this surgeon of the human urethra be correct, contrary to what anatomists have hitherto supposed, no muscular fibres, having a *circular* distribution, capable of giving to it a power of relaxation and contraction, are to be met with, but that its action is wholly dependent upon *longitudinal* fibres, these being short and interwoven with each other, thus surrounding the urethra. The surface of the inner membrane is represented as covered with numerous papillæ, which are the orifices of glands. Irritation of these causes enlargement, and subsequent exudation of coagulable lymph, forming an impediment to the transmission of the urine; in other words, *stricture*.

That microscopic observations, within certain limits, are of great interest and utility, cannot be denied; but that the method of prying into the ultimate conformation of structures by glasses magnifying many hundred times is either very useful or the results very trust-worthy, is matter of reasonable doubt. Setting aside the imagination of the microscopic physiologist, we know that the phenomena are apt to be modified to a very considerable extent by the manner in which the rays of light converge upon the lens; and that objects seen at certain angles are rendered more complicated and obscure than when viewed with less powerful magnifiers. Mr. Andrews throws out these hints as detracting from the confidence with which such researches ought to be received, and then proceeds more directly to comment upon the opinions of Sir Everard.

" Admitting that Sir Everard's statement of the longitudinal fibres is quite correct, it would perfectly accord with the spasmodic action to which the urethra is liable, and the contraction of those fibres would have precisely the same effect on the internal membrane in producing a stricture, as if their arrangement had been circular; for, when the muscular fibres contract in a longitudinal direction, the effect of their action would be to draw the two extremities of the urethra nearer to one another, and in so doing they must necessarily throw the internal membrane into folds, more or less at right angles to the course of the muscular fibres, thereby producing a stricture. But, if we are to look for the formation of permanent stricture from the exudation of coagulable lymph, as described by Sir Everard Home, we should certainly have strictures forming in every part of the canal indiscriminately, and more especially at that part which is the seat of gonorrhœal infection: we should also have coagulable lymph thrown out longitudinally as well as circularly; and moreover we should have no prospect of a permanent cure, or security against a return, by any mode of treatment, since every gonorrhœa, or other cause of violence to which those glands must be so continually exposed, would (agreeably to the known laws of inflammation) be followed with a like deposit of coagulable lymph, and thus produce a return of the complaint.

" The longitudinal, sulcated, or plaited appearance of the internal membrane, which is more or less distinctly seen upon laying open the urethra after death, is easily explained; the urethra being a flexible and distensible, but inelastic tube, surrounded by a loose cellular texture, is naturally disposed to fall into longitudinal plaits during its state of complete relaxation, independent of any muscular action; but it admits of perfect distention in a healthy urethra, during the passing of the urine.

" It is well known that there are certain parts of the canal which are particularly disposed to the formation of stricture; and one of the most usual seats of the disease is at a great distance from that part which is attacked by gonorrhœa,—viz. the membranous part of the urethra, which would of itself be a sufficient refutation of this new theory: and so usually are strictures to be met with in that situation, that I never recollect meeting with one anterior to it without having also found one there; but I have often had a case of single stricture, which has invariably been at that point, and never have met with one beyond it. This, then, warrants the conclusion that the original stricture is formed there, and that others anterior to it are the consequence of certain states of irritation in other parts of the canal, most probably induced by it, and not the consequence of coagulable lymph thrown out, as in the manner before described.

"Assuming, then, that the urethra is lined with a membrane in some respects similar to the membranous lining of other canals, it is found to differ from every other canal in the human body, in having to perform a double office, each requiring a state peculiar to itself; in the one it is in a state of relaxation, capable of great distention at the time the bladder expels the urine through it; in the other (viz. in the erect state of the penis,) it is elongated, and therefore necessarily lessened in its diameter, by which arrangement the semen, in the act of coition, is more readily thrown forward with that projectile force which is requisite to fulfil the intentions of nature.

"This power of elongation and relaxation is, therefore, evidently necessary for the due performance of its natural and healthy functions; but, if abused by their too-frequent repetition, a morbid state of the parts will be the consequence.

"When certain stimuli are applied, which have the power of producing spasm in any part of the canal, the diameter at that point is of necessity lessened during the continuance of the spasm. This, under ordinary circumstances, is only productive of temporary inconvenience, the parts recovering themselves as soon as the spasm subsides: but, if this spasmodic action be frequently reproduced, that portion of the canal which has been most acted on remains permanently contracted, thereby forming the disease in question.

"The disposition to contract or lessen the diameter of the canal, although it is to be met with throughout its whole length, is not found to be equally great in every part, but is more so at two different situations,—viz. immediately behind the bulb, and at four inches and a half from the external orifice, at which places it is naturally narrower than in any other part of the urethra." (P. 4—8.)

Our limits do not admit of our following the author through all the subjects which he investigates, and we therefore pass over the Causes and Symptoms of Stricture, in order to afford space for the practical parts, to which, in all our reviews, it will be observed, we devote the principal share of attention.

With regard to the Treatment of Stricture, Mr. Andrews regards it as of importance to keep in mind that it is a "local affection," and therefore to be removed only by local means; although, at the same time, as it frequently brings on constitutional symptoms of various kinds by sympathy, so these may be relieved by appropriate general remedies. To effect a cure, two modes of treating this complaint are adopted: one palliative, viz. the simple bougie; the other permanent, viz. that armed with caustic. The principal object of the present volume is to "advocate" the latter. Reasoning *a priori* upon the nature of the complaint, he thinks it is more rational to expect that, if by caustic we can destroy the diseased portion of the canal, we shall be more likely to produce lasting relief, than by merely dilating the parts by the common bougie. Perhaps, the principal objection to the method here recommended, is the pain to which it gives rise: this, in irritable constitutions, is frequently of the most acute nature. Where this is the case, the patient ought to be instructed to void his urine immediately after the application.

The objections to the caustic are fully stated, and the comparative advantages of the armed bougie are thus detailed:

"It has been before stated, that the only rational way of explaining the principle on which the simple bougie can act, is by means of dilatation, for which purpose it must be conical in its shape.

"Bougies so constructed, and of such a size as the aperture of the strictures will admit, are to be employed daily, and in most instances ought to be allowed to remain in the urethra for an hour or two at each time they are passed, and their size gradually increased as the aperture becomes enlarged. This process must be continued, until a cylindrical bougie, or flexible gum catheter, of as large a size as the urethra will admit,* can be passed into the bladder. When this is effected, the permanent cure is not even then in all cases to be considered as completed: it will be necessary that the patient should either daily pass a bougie or have one passed for him, as far at least as seven inches, and this for a considerable time, subject to some variation, according as the stricture may or may not have been of long standing, or attended with circumstances of an aggravated nature, which may have brought it into a more or less contracted state; otherwise it will soon be liable to a return.

"It must be recollected that, when once a permanent stricture has been formed, the inner membrane of the urethra becoming elongated at that point, is not restored to its original healthy state by the use of the simple bougie; but, having only been dilated by

* It is here presumed that the external orifice is not itself contracted.

the pressure of it, will readily fall again into contraction soon after the treatment is discontinued, for which a longer or shorter time will be required, depending on the state which the parts were in previous to the treatment, as well as the habits of the patient's life; and the resumption of the bougie will then again become necessary. But when the strictured part has been wholly destroyed by the action of the caustic, the urethra is left free from impediment, and the urine is able to distend it in passing, throughout its whole extent; under which circumstances, it must readily be admitted that the chances of a return are much diminished.

"That this is a fair presumption *a priori*, I think no unprejudiced person can deny; but, in all matters of controversy, facts should be adduced in support of the opinions advanced, I trust that the cases which I shall submit will be found fully to substantiate the proof.

"Much has been said against the caustic, and some have even gone so far as to insist that the cure by that treatment is not more permanent than by the other, and that it is often even productive of fresh obstructions, which are more obstinate and difficult to remove than the original ones; thus endeavouring to make it appear that the remedy is worse than the disease.

"But this is not the fact; and a reference to the only two cases in which I ever noticed a return will be sufficient to disprove the assertion, as in each instance every one of the strictures that returned were found to be at the same points where they had been originally met with." (P. 150—2.)

And again—

"The reason why caustic has disappointed so many practitioners, is, I think, that it is kept too long in contact with the stricture.

"Some persons are in the habit of keeping the caustic in the urethra for a minute, and some even a longer time: this is likely to be productive of an extensive slough, on the detachment of which hemorrhage will very probably ensue.

"We are told by Sir Everard Home, that, as soon as the effect of the caustic becomes sensible to the operator, which is known by the pulsation of the arteries felt between the finger and thumb which grasp the penis, it should be withdrawn. The length of time required for such an effect to take place must necessarily vary in different individuals, depending more especially on the state of the stricture; but the first application should be less than a minute.

"My own observations have for a long time led me to adopt a different rule of practice, by which I consider that I have avoided the risk of hemorrhage to any extent that might otherwise excite alarm. I never keep the caustic in contact with the strictured part, even in the most obstinate cases, more than a few seconds; but my general habit is to touch the stricture lightly, and withdraw the bougie immediately. By this mode many more applications, in a case of long standing, may be necessary, but the pain to the patient is considerably less, and the chances of hemorrhage, or any other unpleasant symptoms arising from it, must obviously be comparatively few.

"By attending to this, and taking care on all occasions that the bougie employed is as large as the external orifice will admit, and the surface of caustic as great as can be safely secured at its extremity, I have no hesitation in saying that a safe and permanent cure may almost always be depended upon, which, it must be universally admitted by every practitioner, cannot be effected by the simple bougie.

"The following cases will show the length of time that those patients have been free from strictures, after their removal by caustic.

Case I.—A gentleman applied to me in 1802, in consequence of a stricture in the urethra. He never had contracted a venereal gonorrhœa, and therefore he considered that the symptoms arose from some complaint in his bladder.

On passing a bougie, I found an obstruction at six inches and a half, to which I applied the caustic, and, after fourteen applications, the unarmed bougie passed on to the bladder.

The symptoms were all removed, the principal of which were a frequent inclination to make water, and involuntary emissions during sleep. In December 1823, twenty-one years after he was considered as cured, I had an opportunity of examining the canal, and found that the largest sized flexible gum catheter passed into the bladder with perfect ease; and, in October 1826, I again introduced the same sized catheter without the slightest impediment.

Case II.—In April 1803, conversing with a gentleman at Madeira on the subject of venereal complaints, he mentioned to me that he was extremely uneasy on account of a discharge from the urethra, which had continued on him from the time of his having contracted a gonorrhœa in August 1802.

For the cure of this, he had used injections nearly one month; at the expiration of that time, the infection was considered to be removed, and what remained was treated as a gleet, with Bals. Capaivæ, &c. &c.

From this account, I did not hesitate to tell him that the continuance of the discharge must be in consequence of a stricture, and, upon making a more minute inquiry into the case, I found that he was subject to painful erections, and involuntary emissions during sleep. My suspicions were confirmed by the passing of a bougie, which met with a stricture at six inches from the external orifice. I now proposed the use of the caustic, to which he readily consented, and on the 20th of April the first application was made. Four applications allowed the bougie to pass on to seven inches, but it required seventeen more before it could be passed into the bladder.

The discharge now completely disappeared, and he has since informed me, that, between the period of his contracting the gonorrhœa and the time of his speaking to me on the subject, he had a discharge of thick glairy mucus from his bladder, which came away mixed with the urine, but, when suffered to remain any time in a vessel, separated, and adhered to the sides of it.

This happened twice, the first time continuing on him nearly a month, the second only a few days; but each attack of it left him suddenly; and, as he did not consider this circumstance connected with the complaint in the urethra, he had purposely omitted taking any notice of it to me before.

Several months after, he contracted another gonorrhœa, of which he was cured, but no return of contraction occurred, as a bougie of the full size passed with ease.

In 1821, this gentleman came to England for some months; and, as he was very desirous of ascertaining whether there had been any disposition to a return of his complaint, he went to my residence at Watford, in order that I might have an opportunity of examining the canal; and I had the satisfaction of finding that a full-sized flexible gum catheter readily passed into the bladder. He then informed me that he had twice contracted a clap since I saw him, which had been cured by injections; so that, if the disposition to contraction still existed, there had been sufficient exciting causes for a return. This was eighteen years after its removal." (P. 161.)

The only other part of Mr. Andrews' work which we can notice relates to a very formidable disease, Stricture of the Œsophagus.

" Strictures in the œsophagus are far from being so common as in the urethra, for two very obvious reasons,—the size of the canal, and the use for which it is intended. However, they are more common than has been generally believed.

" It is only since the use of the lunar caustic has been so beneficially employed in strictures in the urethra, that an attempt has been made to relieve this complaint by the same application.

" Sir Everard Home has given some cases of it in his Treatise on Strictures, and the following have come under my care.

Case I.—I. A—, a Portuguese, gave the following history of his complaint. It first appeared in July 1803, with a considerable hoarseness, after having taken much exercise, and exposed himself suddenly to the cold; the hoarseness continued for several days, more or less troublesome. In October it increased so much, that he was with difficulty understood, accompanied with a dry and painful cough. From this period to February 1804, the cough increased, the expectoration became considerable, and occasionally his respiration was very difficult.

On the 24th of February, he experienced great difficulty in swallowing; and, on the 15th, could not swallow either solids or fluids. In this way he continued for eight days, receiving support from clysters. His medical attendant ordered an application of mercurial ointment and camphor, night and morning, to the outside of the throat.

In the course of eight days, the spasm was so far removed as to enable him to swallow liquids in very small quantities; but, in order to effect this, he was obliged to place his body in an almost horizontal posture, with his head rather elevated.

It was more than six weeks before he could get down any thing that was solid, and this so trifling in quantity, and with so much caution as to his position, that it could not be depended upon as nourishment.

His cough continued increasing; the expectoration was considerable, evidently purulent, and often mixed with blood.

In May 1804, I first saw him: he was in a very weak state, and much reduced. He could then only swallow liquids, and those when in an almost horizontal posture.

At this time I considered his state of health so precarious, that I did not venture to suggest any mode of relieving the throat, but attended wholly to his constitutional symptoms. Various medicines were ordered; and as in a few weeks he gained sufficient strength to be able to walk about the house, the complaint in his throat became an object of attention. I passed a bougie, previously curved, and found it met with a resistance in the œsophagus.

A bougie, armed with caustic, was passed on the following day, and, after eight applications at different intervals, he was enabled to swallow either fluids or solids, in a perfectly erect posture. In two weeks after this he caught cold, and the difficult deglutition again returned, though less violently than on the former occasion. Two applications of the caustic brought every thing right again, and he felt no further impediment to his swallowing till the day of his death, which was suddenly on the 16th of September.

The following were the appearances which I had an opportunity of examining after death. The œsophagus, when laid open from behind, showed the situation of the stricture, a small portion of which still remained; the projection of it appeared like a little shelf: all the other part of the canal was perfectly natural. The lungs were full of tubercles, some of which had suppurated.

Case II.—A young lady, about seventeen years of age, accidentally had a cherry-stone stick in her throat for a considerable time: it was at last brought up by the act of vomiting.

This happened about the year 1787, from which period she was liable to repeated spasmodic affections at that part of the œsophagus, during the act of swallowing. A very small piece of bread touching this part was frequently sufficient to bring it on, so that the food could neither be swallowed nor brought up for several seconds. Within the last few years it had become very distressing, often occurring two or three times during a meal.

In 1804, I first ascertained that these symptoms were produced by a stricture in the œsophagus, as a bougie of a small size could not be passed into the stomach: I therefore applied a caustic bougie, previously curved to it, four or five different times, at the intervals of two or three days. The swallowing was evidently much improved by it; but after one application the throat inflamed and swelled, accompanied with very considerable pain, which extended to the Eustachian tube of the left side; the external ear was also much affected. It became necessary to apply a blister to relieve these symptoms; and, as she was then advanced in pregnancy, I thought it prudent to decline any further continuance of the treatment at that period; since which she has always been in a very delicate state of health, in consequence of a pulmonic affection, so that the treatment could never be continued regularly for a sufficient time to be productive of any permanent advantage; but, whenever the throat was most troublesome, and her other complaints would admit of the application of caustic, it always afforded relief.

Her pulmonic complaints at length getting the better of her constitution, I was anxious to examine the parts after death, and was much satisfied with their appearances, as I thought they afforded considerable information relative to the complaint and its treatment. What appeared of most consequence to notice was the situation of the stricture, which was immediately behind the cricoid cartilage, at which part those that have been examined by Sir Everard Home were formed.

The œsophagus had no appearance of diseased structure in any other part, but was narrower in its whole length than it is usually found to be, owing to the great length of time that the stricture had been formed. The membrane immediately above the stricture was not at all injured by the application of the caustic. The aperture of the stricture was not larger than would admit of a crow's quill being passed through it, and the stricture, after the œsophagus had been slit open from behind, appeared to project from the inner membrane equally all round.

Case III.—A poor woman, about fifty years of age, applied to me in April 1805. Since August 1804, she had experienced great difficulty in swallowing any thing, either fluid or solid, and, within the last ten weeks, could get nothing into the stomach but what was perfectly fluid; any attempt to swallow solids produced vomiting.

On the 18th of April, I applied the caustic, and repeated it on the 19th and 20th. On the 21st, I passed an unarmed bougie into the stomach. On the 22d, 23d, and 25th, I passed a probang with great ease, and, as she swallowed tolerably well, I allowed her to return to her friends in the country.

In May following she came back to me, as she again found great difficulty in swallowing, the act often producing vomiting. On the 11th I applied the caustic, and repeated it on the 12th and 15th, after which she went into the country perfectly relieved.

Our notice of this work has consisted in pointing out the views and practice peculiar to the author, rather than in any attempt to analyse the whole. We can recommend it to our readers as containing much useful information, and many good cases: it is obviously the result of extensive and accurate observation, the only just balance in which to estimate the weight of opinions or the value of practice.

*Observations on the Causes, Symptoms, and Treatment of Derangement of the Mind, founded on an extensive Moral and Medical Practice in the Treatment of Lunatics.* By PAUL SLADE KNIGHT, M.D. formerly a principal Surgeon in the Royal Navy, many years Surgeon of the Lunatic Asylum for the County of Lancaster, &c. &c. *Together with the Particulars of the Sensations and Ideas of a Gentleman during his Mental Alienation, written by himself during his Convalescence.*—8vo. pp. 167. Longman and Co. London, 1827.

THERE are certain topics, the interest of which can never be exhausted; and, whether we approach the consideration of the subject of mental derangement as philanthropists or as physicians, we shall find it constantly presenting to our minds new trains of reflection. The insulated experience of individuals cannot alone ensure the advancement of our knowledge in so extensive a field of inquiry. The co-operation of all, whose opportunities furnish them with the means of investigating the various shades and peculiarities of mental disease, is essentially necessary; and we consider that the communication of the knowledge he obtains, is a moral obligation imposed upon every man to whose care is entrusted an asylum for the insane. Dr. KNIGHT need not, therefore, apprehend the charge of "temerity" for having offered the profession the result of his personal examination of the symptoms of insanity, in the cases of about seven hundred lunatics; "which examinations were carefully made, and very frequently repeated, during the progress of treatment." He assures us that he has studiously endeavoured to avoid all bias in favour of, or against, any mode of practice; and those who wish for a still further confirmation of the facts and inferences stated in the work, are referred to the records of the medical practice in the Lunatic Asylum for the County of Lancaster, which were carefully kept by Dr. Knight, and whence a large portion of the materials for constructing these practical observations have been obtained. The author observes, that he who devotes his talents to the investigation and treatment of mental diseases, "seems to be occupied in a pursuit of doubtful reputation, and to be placed in the lowest cast of the medical profession!" Upon this point we trust he is mistaken. We know of no such invidious distinctions.

Dr. Knight declares his firm conviction that, in every case of deranged intellect, the disease proceeds immediately from corporeal disorder; but yet he has not discovered, in his examinations of the bodies of lunatics, any morbid appearances that are not frequently met with in bodies where insanity had never been manifested. He thinks he has observed greater evidence of excessive vascular action in the brains of insane subjects, than would have remained, under the same system of depletory treatment, in the same subject, had he been sane, and died sane.* We infer, then, that the term "corporeal disorder," employed by the author, goes no further than to imply various functional derangements, with which he presumes the brain may sympathise.

From strong circumstantial evidence, it may be concluded that hereditary predisposition to insanity is the result of peculiarity of structure,—something in the machinery, with which the living principle acts, being defective.

The author draws a strong line of distinction between hereditary and non-hereditary insanity. "Adventitious circumstances sometimes concur, and produce insanity in persons previously, and subsequently, of sound intellect and healthy frames. If the predecessors of such persons have been free from insanity, I have not known a well-authenticated instance of the disorder being transmitted; and therefore I do not consider such event any well-grounded objection to an alliance with the individual: much less do I consider it a well-grounded objection to an alliance with the family." (P. 5.)

The only symptoms of insanity with which Dr. Knight is acquainted, "are a confusion in the intellect, with some degree of perception and consciousness; the confusion being frequently, in the early stage of the disorder, manifested more by actions than words." He employs the terms Insanity and Derangement of the Mind synonimously.

Dr. Esquirol observes, that, in mental derangement, the sense of delicacy is obliterated, and people of the finest previous feelings will deliver themselves up to the most indecent and culpable actions, without the consciousness of impropriety. If this observation is meant to apply to all cases of insanity, it is certainly incorrect. Dr. Knight, on the contrary, has usually met with much propriety and decency of deportment, and he would regard "such general licentiousness as indisputable evidence of bad, very bad moral management." He believes also "that, generally, in the insane the sexual passion is in a state of abeyance, more particularly in man."

* The Appendix to "An Inquiry into the Nature and Origin of Mental Derangement," &c. by Dr. Crichton, contains some interesting particulars of the appearances discovered in the brains of lunatics.

*Of the Moral Causes of Insanity.*—Insanity, like many other diseases, may have for its origin a moral or a physical cause. The author is of opinion, however, that moral impulses very rarely produce insanity; and this is also the case with regard to religious feelings. Of nearly seven hundred cases of insanity that he has sedulously treated, he has only once satisfactorily ascertained that either a religious or a moral cause produced the disorder. Terror, however, claims particular notice: by its violent and sudden action, it frequently produces instantaneous insanity; "and," says Dr. K. "insanity from this cause is the only one resulting from the passions which I have satisfactorily ascertained." Whatever may have been the experience of our author, however, upon this point, it cannot be doubted that mental derangement is the very common consequence of powerful mental impressions, of various kinds.

*Physical Causes and Treatment.*—Dr. Knight has scarcely ever found insanity unaccompanied by one or more corporeal diseases. Neither the statements of the patient nor his friends are to be too implicitly relied on. A lunatic labouring under disease will frequently, when questioned by the physician, deny the existence of every symptom that could lead to its detection. The most careful and guarded examination on the part of the practitioner will consequently be required to enable him to arrive at the true state of the case.

Dr. Knight dwells particularly upon the subject of blood-letting, and quotes many of the most celebrated practitioners of the present day, to confirm his opinion that the use of the lancet is by no means generally demanded. In more than one instance, we have ourselves seen decided mischief arise from the injudicious, and not very uncommon, attempt to cure insanity by copious depletion. In one case, at this moment under our care, the patient, a man of strong health, and with all those appearances which would seem to indicate the propriety of blood-letting, invariably has complained, after the abstraction of blood, by cupping or otherwise, that the throbbing of his temples and pain of the head, of which he had previously complained, were decidedly aggravated. We would not hastily come to a conclusion upon limited experience, but we believe there is but little difference of opinion upon this important subject. Even in the high state of insanity, Dr. Knight "has never seen bleeding lessen the violence of the paroxysm, but, on the contrary, he has seen the excitement augmented by it."

*Idiopathic Insanity.*—Our author is of opinion that insanity is very rarely idiopathic, and, when it is, his experience leads him to conclude that it is manifested in very early life;—it is generally, if not always, incurable. Medicine has appeared to be of very equivocal use in idiopathic insanity, especially if conjoined with epilepsy. When this class of insanity is not accompanied by epilepsy, nor any marked bodily disorder, Dr. Knight is not aware of any rational mode of proceeding that has not moral treatment and the regulation of diet for its basis. "The moral treatment should be commenced by a mild and firm discipline; and, however long habits of insubordination, and its offspring, obstinacy, may have engendered an unruly and troublesome disposition, I have never failed, by steadily availing myself of that authority, which is generally accorded to a stranger placed in a superior station, to enforce such a degree of subordination as to preserve the patient in a tranquil and decorous course of demeanour, so as to impress a transient visitor with the belief that he was a rational and tranquil person." (P. 46.)

In order to counteract what appears to him to be the erroneous conclusions of some physicians, the author gives a few cases illustrative of the effects of certain medicines, under their respective denominations.

*Sedatives.*—Having noticed the confusion which has arisen from the application of this term to drugs of very opposite powers, Dr. Knight proceeds to the consideration of Digitalis, which he informs us "is, in the majority of cases, on its first administration, as decided a stimulus as brandy or geneva!" As this observation is in direct opposition to the opinions generally maintained upon the action of digitalis, we could have wished that the facts which gave rise to it in the mind of our author had been more particularly mentioned. Digitalis has always appeared to us to exert a directly sedative power. By the term sedative power, we imply a *diminution* of the action of the heart and arteries, without any *previous increase**.

"Digitalis, however, never fails, after a few days at least, to reduce the pulse either in force or number, and, in a few instances, both in number and force. Sometimes, however, the pulse loses in power, but gains in velocity, and, when this is the case, I have always found that the medicine was exerting a baneful influence on the constitution; and I earnestly request my junior medical brethren to be exceedingly watchful and cautious of producing this effect. When it is manifested, the vital powers are giving way; and, whilst we are watching for the sedative power of digitalis,—whilst we vainly expect or hope to find the pulse drop from 120 to

* In this we differ from the Reviewer, although we cannot go so far as Dr Knight; who, in our opinion, has expressed himself too strongly.—EDITOR.

a moderate number, our patient will sink into oblivion. I believe Dr. Withering has the honour of being the first to suggest the use of this powerful plant in cases of mental derangement; and, by a due attention to his directions, relative to the state of the fibre especially, the practitioner will very seldom be disappointed. I have uniformly found that this powerful drug exerted a beneficial effect in allaying the maniacal paroxysms and reducing irritability, exactly in proportion as it reduced the pulse, whatever might be the mental action, whether gay or melancholy." (P. 49.)

In a great number of instances, it has been necessary to give digitalis for two or three months successively, generally in small doses three times a-day. By this means the pulse has been kept steady, and the patients have been enabled to enjoy amusements, and to mingle peaceably with others. "I have," says Dr. K. "repeatedly omitted the medicine, and as certainly the insubordinate disposition, and restlessness, and a slight acceleration of the pulse, have followed. On resuming the medicine, the patient has peaceably and cheerfully returned to his usual avocations,—generally labour; and under this treatment has been very much improved in health, and ultimately restored to sanity. I am desirous to direct the particular attention of my reader to these facts, because I know some, (and among them a distinguished physiologist, whose private instructions it was my good fortune to receive,) imagine that digitalis allays excitement only by impairing the vital energy." (P. 52.)

Upon the subject of the administration of Opium to lunatics, M. Esquirol observes, that, "as maniacs sleep badly, opium and other sedatives have been employed, but they are now proscribed, by unanimous consent, as dangerous medicines." In his opinion, regimen and exercise are the only somniferous measures which can be safely recommended, and they are generally successful. It is certainly not the case that sedatives are "proscribed by unanimous consent" in the treatment of the insane in this country, although much caution is required in the administration of opium in such cases. Henbane, Hemlock, and Foxglove, are frequently found very serviceable in allaying irritability. Dr. Knight exhibits the henbane in large doses. He says, "I have also frequently given the Extract of Hyoscyamus, in doses of gr. v. quarta vel sexta quaque horâ, with the effect of tranquillising very restless lunatics; and I have for years been in the frequent practice of giving the same anodyne, in doses of twenty and thirty grains at bedtime, with the most complete success as to procuring rest: nor have I, in any one instance, witnessed any ill effect from its use." (P. 56.)

*Purgatives and Emetics.*—The author has rarely found that lunatics require more powerful medicines of this class than other people.

*Alteratives, &c.*—The blue-pill finds a very warm friend in our author. The following is his opinion of this medicine.

"I beg to state, that, in a very great number of instances in which I have used it in old cases, (for I never have thought it a proper medicine for any recent case,) I have never once witnessed a bad effect; but, on the contrary, there is not a solitary instance where the medicine has not been of some benefit, and many cases where the recovery was chiefly, if not wholly, attributable to it. I have generally conjoined with it either the carbonate of soda, digitalis, or colombo, according as the corporeal ailments seemed to require these remedial means. I am not, however, a devotee to mercury. I should apprehend mischief from its use in most, perhaps in all, recent cases attended with excitement, except as an ingredient in an active purgative." (P. 58.)

"*Of Baths.*—The shower bath frequently relieves the headache and irritability in old cases, when the skin is hot and dry. It may also be advantageously used to allay the irritability and restlessness of some epileptics. By its use I have frequently seen the fit postponed.

"I think the shower bath is not in any other cases peculiarly beneficial.

"The tepid bath, at about 96° Fahrenheit, is very grateful to almost all lunatics, and there are very few cases in which it may not be advantageously used, at least once or twice a week. Besides its influence in promoting a healthy state of the skin, it washes, and secures cleanliness, in some degree at least; and on this account alone is of much value. The cold bath, plunging or otherwise, has appeared to me not so useful as the shower bath.

"*Circular Swing.*—As this machine must be considered more in relation to its physical than its moral action, I shall notice it in this place. It is a mean in the cure of insanity possessing immense power. A patient subjected to its action is speedily affected with giddiness and sickness, and the peristaltic motion of the whole alimentary canal seems to be excited, and in some instances to such degree that the patient vomits, and passes feces in rapid succession and great abundance, along with his urine. I have found the circular swing extremely beneficial in obstinate constipation, and in dyspeptic complaints accompanied with much acid." (P. 60.)

*Of the Moral Treatment of the Insane.*—This very important part of the subject is noticed at some length by the author. The following remarks deserve especial notice.

"It is a great error to pretend to coincide in opinion with the lunatic, acknowledging his pretensions, confirming his opinions, and saying every thing that may be supposed to be pleasant and soothing: fortunate, indeed, will be the result if the effect is not absolutely the reverse. The lunatic, for instance, who has thus been confirmed in his belief of his own sanity, at once becomes restless, irritable, and importunate, although he was previously tranquil and contented. I have known this apparently trivial error in moral management produce raging and ungovernable madness. To me it appears equally absurd, and I know it to be equally prejudicial, to reason (as it is called) or argue with the lunatic, for the purpose of convincing him of his hallucination. Many a well-meaning person, confiding in the cleverness of his reasoning faculty, may be seen combating the false perceptions of the peaceable lunatic; for it is with the peaceable only that these sage persons enter the lists: they never venture to engage with the turbulent or the excited, although frequently the saner of the two. The peaceable lunatic becomes at first a tranquil and willing auditor; till, finding his understanding insulted, by the evidence of his senses being either absolutely denied or boldly questioned, he becomes indignant at the barefaced assurance that would impose on him as truth that which the evidence of *his senses*, perhaps anxiously and repeatedly examined, tells him to be false. It will be found most prudent, most conducive to the patient's recovery, to permit the accuracy of these insane perceptions and morbid ideas to go unquestioned, and perfectly unheeded,—to carry the lunatic's attention to a very different subject,—and to fix it, as much as possible, on that which has no relation to the hallucination." (P. 70.)

Coercion in a dark room is considered generally sufficient even for very unruly lunatics. It is of the utmost importance that patients should be taught that resistance is useless; for insufficiency, as well as harshness and severity, begets a spirit of resistance, and has a direct tendency to excite furious mania. "It ought to be a law in the treatment of the insane, that all restraint is improper which is not imposed either to prevent the patient from injuring himself or others; and then the moral treatment which preceded the coercion should be jealously inquired into. For I fear it will be sometimes found, even in the best-regulated establishments, that the necessity for coercion has arisen out of some mismanagement in the prior moral treatment." (P. 74.)

Various amusements are pointed out proper to relieve the tedious hours of the patient labouring under insanity. Dr. Knight observes, however, "that the insane should never be encouraged to write." It is also recommended that lunatics should not be permitted to stroll about alone. If they are excited, they will take too violent exercise, and, if depressed, not enough; and the mind on these occasions seems peculiarly prone to indulge in its insane reveries.

*Labour* of some kinds should, if possible, be allotted to all lunatics. Some stated task should be imposed, which they should, if possible, be made to perform. Lunatics should not be permitted to idle away weeks, months, years, in the apartments or yards of their abodes. This is highly reprehensible and disgusting, and a disgrace to all concerned whenever it is permitted. The use of the common wheelbarrow is suggested as the best employment.

*Religion.*—The author would by no means debar the insane from the exercise of religious duties. We confess, however, we doubt the propriety of selecting "a dissenting clergyman, who was *generally well enough* to give a discourse that at least gratified many of his hearers." For such an office, a person should be chosen with whom the patients are not in the habit of living, and for whom they would feel more respect than for one of their companions in confinement. As far as we have seen of lunatics, we have generally observed among them a determined objection to the counsel or advice of each other. On examining sixteen letters published by the Committee of the New Bethlem in London for 1817, and received by the committee from various physicians and superintendents of Lunatic Asylums, fifteen speak favourably of the effects of religious instruction in their respective establishments; and in the other no religious instruction had been resorted to.

*Music.*—The author has never witnessed any bad effects from indulging the insane with this agreeable amusement. At the same time, he would not indiscriminately allow it to all lunatics. He has never ventured to try it on the excited and recent cases, nor does he recommend the experiment.

*On the Method of securing Lunatics.*—Several plates are given, representing different modes of confining patients. They are such as we believe are generally employed, and appear well adapted to secure the lunatic from inflicting injury upon himself or others.

Some tables of classification are introduced, by which the results of old and recent cases of insanity are shown.

The volume concludes with an interesting account of the various sensations felt by a person deprived of reason by a fever, with a description of the scenes in which he thought himself employed. This account is dedicated to Dr. Knight by the patient, on his recovery.

We can recommend the present work to those who require a concise and practical view of the subject on which it treats. The author has, we think judiciously, avoided all those metaphysical disquisitions which some of our writers on Mental

Derangement have been too fond of indulging in. He has been satisfied with the less imposing, but more useful, plan of stating the results of his own experience.

Throughout the work we observe the expression of a spirit of sympathy and benevolence for the unfortunate sufferings of those who were confided to his care, which would induce us to believe that Dr. Knight was peculiarly well fitted for the responsible situation he held for many years in an extensive lunatic asylum.

---

